# Bibliographical Notices

**Published:** 1843-04-01

**Authors:** 


### Bibliographical Notices.

[*Continued from page* 484.]

On the Chemical Discrimination of Vesical Calculi. By *E. A. Scharling*, A.A. L.L.M. Professor of Chemistry in the University of Copenhagen. Translated from the Latin, &c. By *S. Elliott Hoskins*, M.D. With Plates. London. J. Churchill. 1842.

The very numerous works which have issued from the press within the last few years on the very important subject of calculous disorders, would appear to preclude the necessity of an additional one within so short a period. There are certain peculiarities in Dr. Scharling's work, however, sufficient to recommend it to the perusal of those who wish to engage in chemical researches on the properties of calculi; his mode of classifying calculi is novel, and such as, in our opinion, contributes not a little to render their examination easy; and this too is we believe the only work in which rules for separating the usual mixtures of calculi from each other, are clearly laid down, rules moreover which come recommended to us as being deduced from actual analyses.

We shall now proceed to present to our readers a very succinct analysis of the leading parts of the work, commencing with the

### Discrimination of Calculi.

*Physical Characters of Calculi.*

*Form.*—The most common forms of vesical calculi are the spherical, oblong and the amygdaloid, but somewhat compressed laterally, so as to give them the shape of pebbles. They are sometimes ovoid.

Many specimens are irregularly-shaped in consequence of the increment taking place more at one side or at one extremity, than at the other.

*Size.*—Urinary deposits are generally designated as sand or gravel, until they exceed two or three lines in diameter. Those worthy of the name of calculi vary in size from that of a pin's head to that of a turkey's egg, and beyond; some weigh a few grains, others several pounds.

*Specific Gravity.*—This is stated to vary from 1·213 to 1·976.

*Surface.*—The surfaces of calculi are frequently so characteristic of their composition, that mere inspection will often suffice to enable us to recognize the constitution of at least the external layer. Witness the rugged surface of the mulberry calculus. Uric-acid calculi are either smooth and even as to surface, or finely tuberculated, the tubercles not bearing any resemblance to the asperities of the mulberry calculus.

*Colour.*—Among the external features of calculi none are more distinctive than colour.

1st. A tawny-yellow, or fawn-colour running through various shades into reddish-brown, indicates the uric acid series. 2nd. The oxalate of lime formation is denoted by its dull purple colour inclining to brownish-black. 3rd. The phosphates and carbonates are either purely white, or partake of a pale yellow or buff-tint. These observations relate solely to the colour of the external surface.

*Odour.*—Fourcroy it was who compared the odour of calculi, when sawn or rasped, to that of bone or ivory, and that the spermaceti odour given out by mulberry calculi, when abraded, was characteristic of that variety.

### CLASSIFICATION OF CALCULI.

Various methods of classifying urinary calculi have been proposed; some founded on their sensible properties, and others on their chemical composition. Our author conceives the most natural classification to be that in which not only the whole of the constituents are enumerated, but also the order of their succession. In order to facilitate their identification it is also necessary to class them according to their chemical properties. In his classification he has adopted both these plans.

*Table of Vesical Calculi.*

| | *Nucleus.* | *Principal Mass.* | *Cortex.* | |
|---|---|---|---|---|
| 1 | Uric acid ............ | Uric acid ............ | Uric acid ........ | 32 |
| 2 | ,, ,, | Uric acid and urate of ammon. alternat. .... | Urate of ammonia.. .. | 1 |
| 3 | ,, ,, | Uric acid ............ | Phosphate of magnesia | 13 |
| 4 | Urate of ammonia...... | Urate of ammonia...... | ................. | 3 |
| 5 | Urate of soda.......... | Uric acid ............ | ................. | 1 |
| 6 | Urate of lime.......... | Urate and oxalate of lime and urate of ammonia | ........ ........ | 1 |
| 7 | Oxalate of lime........ | Uric acid ............ | ................. | 3 |
| 8 | ,, ,, | Uric acid and phosphates mixed.............. | ................. | 1 |
| 9 | Triple phosphate ...... | Triple phosphate ...... | ................. | 8 |
| 10 | Fusible matter ........ | Fusible matter ........ | ................. | 6 |
| 11 | Urate and oxalate of lime | Oxalate of lime........ | ................. | 8 |
| 12 | ,, ,, | ,, ,, | Phosphates ........ | 13 |
| 13 | ,, ,, | Oxalate and urate of lime alternating.......... | Phosphates ........ | 3 |
| 14 | Urates of ammonia & lime | Urates of ammonia and lime alternating...... | ................. | 2 |
| 15 | Uric acid and urate of ammonia .......... | Urate of ammonia and phosphates mixed.... | ................. | 2 |
| 16 | Uric acid & urate of lime, | Oxalate of lime........ | Phosphates ........ | 2 |
| 17 | ,, ,, | Phosphate ............ | ................. | 2 |
| 18 | Uric acid, urate and oxalate of lime ........ | Uric acid ............ | ................. | 27 |
| 19 | Uric acid, urate of ammonia and oxal. lime.. | Phosphates .......... | ................. | 2 |
| 20 | Uric acid, urate and oxalate of lime ........ | Uric acid ............ | Phosphates ........ | 17 |
| 21 | Silica................ | Silica................ | ................. | 1 |
| 22 | ,, | Carbonate of lime..... .. | ................. | 1 |
| 23 | Foreign materials...... | Phosphate of lime...... | ................. | 2 |
| 24 | No nucleus .......... | Phosphate and carbonate of lime ............ | ................. | 1 |
| 25 | ,, ,, | Phosphates .......... | ................. | 2 |
| 26 | ,, ,, | Fibrinous matter and phosphates ........ | ................. | 1 |
| | | | Sum total of calculi.. | 155 |

Thus, in 94 the principal mass is uric acid = 60 per cent. = $1 : 1\frac{2}{3}$

23 ,, ,, oxalate of lime = 15 ,, = $1 : 6\frac{2}{3}$

25 ,, ,, phosphates = 16 ,, = $1 : 6\frac{1}{4}$

In the above table the ingredients forming the nucleus have been arranged in the first column; in the second, those constituting the body or principal mass, and in the third, the material of which the cortex, crust, or external covering, is composed. When the latter coincides in composition with that of the principal mass, a blank space has been left.

The classification proposed by our author is founded on the changes which are produced on calculous concretions by exposing them to a high temperature.

The effect first produced on a fragment exposed to the blow-pipe flame is, that it becomes charred, owing to the combustion of the animal matter calculi always contain.

If the heat be continued, or augmented, one of the following conditions is fulfilled,—

1st. The fragment gradually consumes, leaving no residuum or a very slight one.

2d. The fragment does not consume; it either whitens after being charred, and remains unchanged, or it fuses into a head.

3d. The fragment partially consumes, leaving a considerable proportion of residuum.

These three differences naturally suggest the division of calculi into three classes.

Class 1st.—*Combustible Calculi,* which consume on being sufficiently heated, leaving a very minute residuum.

Class 2d.—*Non-combustible Calculi,* which do *not* dissipate under calcination.

Class 3d.—*Partially Combustible Calculi,* which, after combustion, leave a considerable proportion of residuum.

The first class includes the following orders:—

A. *Uric acid.*

B. *Uric oxide.*

C. *Cystine.*

D. *Combinations of the three preceding, with each other, or with other combustible materials, as ammonia,* &c.

The second class includes the following orders:—

A. *Phosphates.*

B. *Carbonates.*

C. *Silica.*

D. *Mixtures of the preceding.*

The third class includes the following orders, namely:—Compound calculi, formed by the combination of the species contained in the two former classes.

A. *The residuum consistinq of the phosphates.*

B. The residuum, consisting of carbonates or caustic bases.

C. The residuum, consisting of phosphates, with carbonate or caustic bases.

D. The residuum, consisting of accidental ingredients, either alone, or mixed with the foregoing.

### *On the Origin and Increment of Calculi.*

The mechanical formation of calculi evidently depends on the gradual deposition of minute particles, either amorphous or crystalline, on some solid molecule existing in the bladder. This mass may be a small calculus which has descended from the kidneys, or some foreign substance accidentally present in the bladder.

When instruments are allowed to remain for any time in the bladder, they become coated with a deposit from the urine, which deposit generally consists of the phosphates. Other substances resident in the bladder, also acquire this sort of incrustation. As similar phenomena occur when a solid substance is placed in concentrated saline solutions, we may infer that urinary concretions are formed by a similar process of deposition. Prout, and others, however, entertain a somewhat different opinion: they consider, that precipitation does not occur until the foreign substance has produced so much irritation as to affect the kidney sympathetically, so as to cause secretion of urine supersaturable with precipitable salts.

Berzelius considers that urinary concretions form under the following circumstances; first owing to the kidneys secreting an over-abundance of the salts sparingly soluble in urine. Secondly, the absence of a sufficient quantity of free acid to retain the ordinary proportion of phosphatic salts in solution. Lastly, a morbid tendency in the kidney to secrete matter not naturally contained in the urine, such as oxalate of lime, which being insoluble, precipitates as soon as formed. Any of these conditions occurring, deposits, he says, take place, which are either carried out of the system, in the form of gravel, or become consolidated and remain for a time in the pelvis of the kidney, from whence, in all probability, they ultimately make their way into the bladder.

When renal calculi have descended into the bladder, unless speedily expelled, they act as foreign bodies, affording a foundation for subsequent deposition. The degree of rapidity with which precipitation takes place, depends on various causes. Among these may be enumerated the envelopment of the nucleus, in albumen, blood, mucus, pus, or any other organic matter that chances to be present in sufficient quantity. These form a villous coat around the solid material, and their flocculi arrest, entangle, and ultimately determine the crystallisation of the more insoluble ingredients of the urine. This explanation will go far to account for the animal matter contained in all calculi, the presence of which adds so much to the difficulty of distinguishing their constituents.

These organic substances may be considered as a kind of cement which binds calculous constituents together; and not only favours their increase, but in very many instances lays the foundation for precipitation.

*Solvents for the Stone.*—Various solvents have been at different times recommended for the solution of gravel and calculus. And first, with respect to uric acid, the most frequent of any of these concretions. Uric acid requires ten thousand parts of water for its solution; whilst its combinations with the fixed or volatile bases are readily soluble in 480 parts of water. Hence the carbonates of soda and potash are available as solvents for this species of concretion. The solvent powers of these salts are not confined to urates alone. They act equally well on oxalate of lime and phosphatic calculi.

On digesting oxalate of lime in a solution of the alkaline carbonates, an interchange of principles takes place, carbonate of lime, and oxalates of soda or potash result. The two latter salts are soluble in water alone, and the former in water impregnated with carbonic acid. The phosphates are decomposed by the alkaline carbonates, especially the neutral phosphate of lime; more especially by using the bicarbonates. To this circumstance may be attributed the beneficial effects produced by certain mineral waters. Lime-water and carbonate of lime are sometimes useful. These constitute the chief ingredient in Stephens' remedy. In cases where alkalies have failed to relieve, or where they disagree with the digestive organs, carbonate of magnesia has often been employed with advantage—the calcined earth has also been given.

The direct administration of the alkaline carbonates, is not the only, and not always the best, way of employing them. For the neutral salts formed by vegetable acids become converted by a vital process into carbonates during their passage through the system. Caustic potash, as being a more powerful solvent of uric acid than the carbonate, is frequently beneficial. Borax also has been found capable of dissolving uric acid, even in larger quantity than the carbonates of either potash or soda.

Boettiger has experimented on this subject, and finds that four grains of biborate of soda, dissolved in an ounce of water at 98° F. speedily and entirely dissolves a large proportion of uric acid. Our author has found this to accord with his own experience. He employed a solution composed of twenty-four grains of borax to six ounces of water, and found that it dissolved nine grains of uric acid, a part of which was re-deposited at the end of a few hours. He then tried the acetate of soda, and found it less efficient as a solvent than the biborate. Boettiger considers that borax would act more efficiently if used as an injection; our author entertains the same opinion with respect to the acetate of soda, if used as a solvent for phosphatic calculi. The effects of acetate of potash are precisely similar to those of soda.

For the solution of earthy calculi various vegetable acids have been employed, especially the lactic, malic, and citric, on account of the solubility of the salts they form with lime. Tartaric acid also has been used, but with little prospect of success, from the well-known insolubility of tartrate of lime. As factitious waters, such as soda-water, are likely to owe their effects rather to the alkali than to the carbonic acid they contain, the soda-water, as usually prepared, by merely condensing carbonic acid in water, is less likely to be available than an effervescing draught made with bicarbonate of soda and a vegetable acid.

Various arguments have been advanced for and against the utility of the alkaline treatment in calculous disorders. Some warmly recommend it, whilst others aver, that alkalies, far from being beneficial, are decidedly injurious, tending, rather, to increase than diminish any existing concretion.

Our author, however, is satisfied, from his own experience, of the efficacy of solvents in calculous affections; he has distinctly seen the surfaces of calculi erroded and in a cellular state from the effects of chemical agents.

Dr. Scharling mentions, that it has been frequently proposed, instead of taking chemical solvents into the stomach, to inject them into the bladder, so as to place them in immediate contact with the stone. The difficulty is to discover a solvent which, whilst it acts energetically on the stone, should exercise no injurious effect on the animal tissues. He does not seem to be aware of Sir B. Brodie's successful employment of very dilute nitric acid as a lithontriptic injection, a practice which other English surgeons have adopted in various cases.

We shall here close our notice of this little work, not without recommending it to the medical practitioner as a useful guide in the chemical discrimination and recognition of vesical calculi.

---

### The Bengal Dispensatory, and Companion to the Pharmacopœia. By *W. B. O'Shaughknessy*, M.D. Assistant-Surgeon, Bengal Army.

The publication of this work is an era in the medical literature of the East; and its influence will even be felt in Europe.

The Editor had a new field open to him, and has displayed much diligent research in the cultivation of it. He apparently complains—that an order of the Indian Government compelled him to publish this edition before his investigations were complete. We can sympathise with him in his feelings on being arrested in the course of enquiry which he was pursuing. We would, if it were necessary, acknowledge the claim to lenient criticism; but we prefer viewing this edition as a first instalment of what is to come, and we cannot very strongly condemn the impatience of a government anxious to diffuse the benefits of the publication; and to engage in the enquiry the attention and observation of their many other medical officers. We know the uncertainty of human efforts too well to lament the appearance of this work—prematurely for the Editor's wishes—but not so, we think, for a fair amount of benefit and credit.

The investigations which he and others have been making, have not been unknown to us,—we have transcribed the facts as they have appeared from time to time in the Indian Journals.

The efficacy of narcotine as a substitute for quinine is familiar; but the scarcity of the remedy, and the great expense of preparing it in this country, make it inapplicable.

The alcoholic extract of Gungah (Cannabis Indica), has been made extensively known through the medium of this Journal, and has excited much attention. It has been successfully employed, and its use is rapidly increasing. It is spoken of as an anodyne and sedative superior to opium, possessing those qualities equally, yet being devoid of the unpleasant after-effects. It has been successfully given for the relief of cough in phthisis,—of rheumatic pain,—of the delirium in low typhus fever,—to promote sleep in persons who have ceased to derive advantage from opium,—for the relief of hooping-cough,—chorea, and many other spasmodic diseases in this country. The success which has followed its use in all these cases, has given surprise, and the confidence in the drug is increasing.

The necessity of having a special Pharmacopœia and Dispensatory for India, is well set forth. First, "The British Pharmacopœia, containing a great number of indigenous plants, which do not grow in India, or the limitrophe countries, but for which Indian substitutes may be found." This argument applies to others, as well as to the limits of this country.

The Poppy Oil is introduced as a substitute for that of the Olive, which is an article of importation from England. The sweet oil of the Sesamum Orientale, which is universally cultivated in the East, and the very mildly agreeably tasted oil of the Arachis Hypogœa (Earth Pistachio), which has lately been introduced and is now much cultivated about Calcutta, are also recommended both for culinary purposes and pharmaceutical use, instead of the abundant product of the coasts of the Mediterranean—the staple of Italy, Spain, and the South of France.

Conium Maculatum is retained in this work; yet its application, after a voyage to India, and the length of time it must necessarily be kept in the apothecary's hands, previous to its use, must leave great doubt of its efficacy. Indeed, the diseases in which it is usually employed in temperate climates are not of frequent occurrence in the East. The Editor manifestly contemplates the introduction of the Conia, or the alcoholic extract proposed by Dr. Christison, as its most convenient form.

Secondly,—"The British Pharmacopœia do not contain numerous plants indigenous in India, of proved utility as remedial agents."

*Thalictrum foliosum, Wall.* Offi. Root.

It has been used in the Hospital of the Medical College in several cases of ague, and as a tonic in the convalescence from acute diseases.

Gr. v. of the powder, or gr. ij. of the watery extract given thrice daily, have in some cases prevented, and in several moderated, the accession of fever, and at the same time acted gently on the bowels. The only sensations experienced were warmth at the epigastrium, and a general comfortable feeling. The bruised root having been given to large dogs, in doses of from gr. x, to ʒij, no particular effects were observed.

It deserves extensive trial, and promises to succeed well as a febrifuge of some power, and a tonic aperient of considerable value.

*Coptis Tecta, Wall.* Offi. The Root.

The Coptis Tecta has found its way through the drug-shops of Bengal, and is even occasionally exposed for sale in the upper provinces; it brings a high price, and is deemed a tonic remedy of the greatest value. It was used extensively in the General Hospital by the late Mr. Twining, who reported that its influence in restoring appetite, and increasing the digestive powers, was very remarkable, and that it might be said to possess all the properties of our best bitter tonics.

Further trials in the College Hospital were equally satisfactory. It did not seem to exercise any febrifuge virtue, but under its influence several patients recovering from acute diseases, manifestly, and very rapidly, improved in strength. The dose was from gr. v, to gr. x, of the powder, or ℥j, of the infusion thrice daily.

*Coculus cordifolius.* (*Vern. Guluncha.*) Offi. The Root and Stems.

This is one of the most common and valuable plants in India. The fresh root is used extensively by the natives of Bengal, being mixed with sour rice gruel and sugar, for the cure of heat of urine in gonorrhœa. The stem, roots, and leaves are bitter, and are much used, in the form of decoction, as a tonic in convalescence from fevers and acute diseases generally.

In several trials, made at the College Hospital, the Guluncha was found to be a very useful tonic, but we could scarcely attribute to it any very decided febrifuge effect. The decoction was of great use in the treatment of several cases of chronic rheumatism, and of secondary venereal affections. Its action is decidedly diuretic and tonic in a very high degree.

*Berberis Lycium.* Offi. Root and Bark.

A watery extract, called Rusot, is prepared in the hilly districts of India. Dr. Royle has very ably traced the history of this substance, and identified it perfectly with the celebrated λοχιον 'ινδιχον of Dioscorides.

The trials instituted on the medicinal virtues of rusot, have led us to some satisfactory results.

Rusot is best given as a febrifuge in half-drachm doses, diffused through water, and repeated thrice or still more frequently daily. It occasions a feeling of agreeable warmth at the epigastrium, increases appetite, promotes digestion, and acts as a very gentle but certain aperient. The skin is invariably moist during its operation.

In more than thirty cases of tertian ague, (several complicated with spleen), we have succeeded in checking the fever, on an average, within three days after commencing the rusot.

In eight cases of quartan ague, six were cured.

The cases of common quotidian, successfully treated in this manner, were so numerous that they were not recorded. In no instance was headache or constipation produced, but we have seen rusot exasperate the symptoms of chronic dysentery, or hepatitis, when complicated with ague.

On the whole, we deem rusot a most important accession to our Materia Medica, and worthy of being substituted in a multitude of cases for cinchona bark. Its aperient action renders it especially valuable.

*Dipterocarpus lævis.* Hamilt.

The close resemblance in physical and chemical properties which this bears to copaiba balsam, led to the institution of an extensive set of experiments by the Editor on the medicinal effects of the former in the treatment of gonorrhœa. The results which have been laid before the profession, and which have been confirmed by trials made by other practitioners, lead to the conclusion that, in the treatment of gonorrhœa, gleet, and similar affections of the urinary organs, the essential oil of grugun is nearly equal in efficacy to the South American drug.

The essential oil may be given in 10 to 20 drop doses, and repeated thrice, or still more frequently, daily. It generally causes a sensation of warmth at the epigastrium, eructation, and sometimes slight purging. It communicates a strong smell of turpentine to the urine, which it increases remarkably in quantity. Some obstinate cases of chronic gonorrhœa and gleet, which had long resisted copaiba and cubebs, have been cured by this remedy in the course of the experiments alluded to.

*Azadirachta Indica.* Juss.

Every part of this tree, especially the bark, is bitter; the bark is also astringent, the leaves are bitter, and very nauseous; from the ripe pericarp of the fruit a very bitter fixed oil is expressed; the trunk of the tree yields gum, and the young trees, when tapped, yield a saccharine sap or toddy, capable of undergoing the vinous fermentation.

The oil is thought to be anthelmintic, and is applied externally to foul ulcers, and used as a liniment in rheumatic and spasmodic affections, and in headaches from exposure to the sun. The wine or toddy is supposed to be stomachic, the dose being an ounce and a-half every morning. Dr. White, of Bombay, used the bark of this tree as a substitute for cinchona, and with nearly equal success as a febrifuge remedy. Mr. Skipton has related a case of hysteria in which a decoction of the leaves was used with the best effect.

*Calotropis Gigantea.* Mudar.

The powdered bark of the root, in doses of from half a drachm to one drachm, proves emetic after an interval of from twenty minutes to an hour, generally causing much nausea, and in about one case in every three inducing a cathartic operation. In doses of from two to five grains taken every half-hour, it proves nauseant, powerfully diaphoretic, and after several doses gently cathartic.

*Pharbitis Cœrulea.* Wall.

The powdered seeds in doses of thirty to forty grains act as a quick, safe, and pleasant cathartic. We have made this seed the subject of numerous experiments. In 100 cases in which it was given under our direction in the Police Hospital of Calcutta, it proved purgative in 94, occasioned vomiting in five, and griping but in fifteen, and produced, on an average, five stools within 2½ hours; the operation generally commenced in an hour, and in these experiments was never delayed beyond four hours.

The alcoholic extract, which consists of resin and oil, is of excellent pilular consistence, and keeps for several months. In ten-grain doses it produces all the effects of jalap, with certainty and speed: the taste is scarcely perceptible. We have thus a remedy of unparalleled cheapness, perfectly equal to jalap as a cathartic, superior to it in portability and flavour, and occurring in all parts of India.

3rdly. "The pharmaceutical processes in the British Pharmacopœia, especially with reference to distillation, evaporation, making of tinctures and alcoholic extracts, and many other articles, such as ethers, are modified so as to meet the price of labour and spirit, and the operation of excise laws; restrictions are not felt in India, where alcohol of the pharmaceutical strength (sp. gr. 835) may be had for 2s. 8d. the gallon of four imperial quarts—a circumstance which affords immense facility to a multitude of processes. The cheapness of spirits may moreover enable the editors of the Pharmacopœia to effect decided practical improvement in the preparation of vegetable proximate principles, such as quinine and morphine—as well as in the extracts, tinctures, &c. It will enable them also to give efficient directions, and point out various plans for prosecuting analytical researches after new vegetable remedies. Much will be done towards reducing the prices of a great number of medicines essential to clinical practice.

4thly. In the chemical preparations, especially those of the alkaline and metallic salts, perfectly new directions would be essentially necessary in many instances.

Sulphate of magnesia being an abundant natural product in England, the calcined and carbonate of the same base are manufactured from that article. In India however our native druggists should receive instruction how to produce magnesia from the residue after the preparation of common salt, from the magnesian limestone of Sylhet and the Himalaya, and from the magnesite of Madras.

Potash is obtained in Canada by the burning of the forest timber; in Ireland from the fern; in France, Italy, and along the Rhenish wine-districts from cream of tartar; in India with most economy from nitre;—in each case a totally different process is required for its extraction, purification, and adaptation to medicinal use.

Soda is obtained by the Spaniard most easily from barilla by mere washing; by the Scot from kelp, by a much more laborious and unproductive process; in France, and latterly in England, it is prepared from common salt, which is first decomposed by sulphuric acid, and then the resulting sulphate of soda subjected to another process, which changes it into the carbonate. In Bengal and Mysore the soil in many places is so impregnated with the alkali, that incineration and washing extract from 35 to 50 per cent. of fine soda. Ammonia and its compounds would also require considerable alteration. Processes would be necessary for the reduction of several mercurial compounds from the Chinese cinnabar, and the crude chloride of the bazaars.

With reference to the antimonial preparations, methods should be pointed out of distinguishing between the sulphurets of lead and antimony, two substances common and cheap in the bazaars, and resembling each other so strongly that they are constantly confounded even by the drug dealers themselves.

5thly. An urgent necessity exists for alteration of the nomenclature of the British Pharmacopœia, to meet the circumstances, and degree of education of the natives of India. In a territory where not one native understands the Latin language, it would be monstrous pedantry to retain exclusively the Latin names of the present Pharmacopœia. Of the vegetable articles of the Materia Medica nine-tenths are of Eastern origin, and have familiar names in the Bengali, Hindoostani, or English tongues. With the natives of the country there is no need of any intimate acquaintance with the scientific names. The object is to meet the wants of an immense, an ignorant, and poor population, among whom medical science is but beginning to establish itself; among whom, were we to distribute a full proportion of well-educated practitioners, their acquirements must be almost useless, owing to the high prices of all medicinal preparations sold by the private European establishments in Calcutta, Cawnpore, and Meerut—the only localities of Bengal and Upper India where European apothecaries have settled. Little competition existing, the prices of the most indispensable articles of medicine are fixed at such a rate that rich natives will not, the poor cannot, avail themselves of the remedies which medical science has pointed out.

The intention is to promote the supply of competent practitioners and druggists—to furnish them with instruction by which they may successfully perform all the operations and processes which pharmacy requires—to present them with a list of remedies, the natural history of which has been investigated, and the medical virtue determined by adequate authority or clinical observation;—to specify the precautions under which a medicine, having dangerous as well as remedial properties, should be administered—to warn against the use of dangerous remedies in good popular repute—and to give a few simple directions to guide the purchaser or collector by the natural characters of the drug, and its usual price in the market. It is to be a hand-book to the inexperienced practitioner, where all are inexperienced—the "Apothecary made easy," and adapted to the most limited comprehension for the benefit of our black brethren of Upper India and Bengal.

In future editions we shall expect to see the editor exercise a bolder judgment: This is a careful and judicious compilation from our own Pharmacopœia, with additions from sources peculiar to India. The author has not forgotten his British education, and he has not therefore conformed as much as he will hereafter do, to the peculiarities of the Indians. He has also felt that he wrote for British critics, and this feeling, while it has made him correct, has restrained him from making the most of the clear field which lay before him. No doubt correctness is of the highest value in scientific writings; but it ceases to be a virtue when the correctness of compilation checks the expression of correct observation. The quick energy of thought and perception which Dr. O'Shaughnessy has shown, in what he has hitherto done, calls for our utmost praise. The eye that detected the false Angustura bark, in that about to be administered to the patients in the hospital, and identified it with the bark of strychnos nux vomica;—that detected the very valuable antispasmodic effects of gunjah, which had been passed over unnoticed by other Europeans for a century;—and that adopted as a medicinal agent, the popular, but hitherto unnoticed purgative, pharbitis, needs little check from the many eyes that look for years, yet see not.

---

### CONCISE HISTORICAL SKETCH OF THE PROGRESS OF PHARMACY IN GREAT BRITAIN. By *Jacob Bell.*

PHARMACY was originally in the hands of the physicians, who prepared their medicines themselves, or superintended the preparation of them. The science of medicine was then but little understood, and was often confounded with witchcraft; in fact, the Greek word Φάρμακεω signifies either to practise witchcraft or to use medicines.

The first act of parliament relating to the medical profession was passed in the year 1511; by this the faculty of medicine was vested in one body of practitioners who exercised medicine, surgery, and pharmacy. The physician's assistants were styled apothecaries, and these soon began to transact business on their own account.

In the year 1518, the College of Physicians was established. In 1540, their powers were increased, and they had the right of entering the houses of the apothecaries in London, examining the drugs, and destroying such as they deemed unfit for use. In the same year the barbers and surgeons were united into one company, but the surgeons were prohibited from shaving, and the barbers were restricted from performing surgical operations.

In 1695, the question was tried as to whether surgeons were entitled to administer medicines internally, when it was decided "that no surgeon, as a surgeon, might practise physic for any disease."

It is uncertain at what time the physicians gave up the practice of preparing their own medicines, but it appears that in 1617 the apothecaries obtained a charter constituting them a company by themselves wholly to be employed in the business of pharmacy. It was enacted at the same time that no grocer should keep an apothecary's shop, and that no surgeon should sell medicines.

The first Pharmacopœia was published by the College of Physicians in the year 1618. This was the first step towards the improvements which have since obtained in pharmacy. This was however a very imperfect production, the compounds chiefly employed being either empirical nostrums, or mixtures of great numbers of substances, without any reference to scientific principles. Thus the Mithris was a compound of seventy-two ingredients, and many others were equally as absurd.

Culpeper, in his translation of the Pharmacopœia, gives a list of the remedies derived from the animal kingdom, contained in the Pharmacopœia. The following will serve as a specimen, with Culpeper's remarks in parentheses.

" The fat, grease, or suet of a duck, goose, eel, bore, heron, thymallos, *(if you know where to get it,)* dog, capon, bever, wild-cat, stork, hedge-hog, hen, man, lyon, hare, kite, or jack, *(if they have any fat I am persuaded 'tis worth twelvepence the grain,)* wolf, mouse of the mountain, *(if you can catch one,)* ........ album græcum, east and west bezoar, stone taken out of a man's bladder, viper's flesh, the brain of hares and sparrows, the rennet of a lamb, kid, hare, and a calf and horse too. *(They should have put the rennet of an ass to make medicine for their addle brains.)* The excrement of a goose, of a dog, of a goat, of pigeons, of a stone horse, of swallows, of men, of women, of mice," &c. &c.

But though Culpeper justly ridicules the College of Physicians for inserting such wretched trash, some of his own remedies are by no means first-rate; thus he says—" The head of a cole-black cat being burnt to ashes in a new pot, and some of the ashes blown into the eye every day, helps such as have a skin growing over their sight."

About this time chemists begin to be mentioned, as a class of men who prepared, for the use of the apothecaries, the "chemical medicines," or those of mineral origin, and which were subjected to fire.

In the year 1671, however, the Society of Apothecaries, which had continued to prosper, established a chemical laboratory, in addition to the dispensary; thus uniting in one establishment the preparation of chemicals and galenicals. This institution was at first on a small scale, but the superior quality of the articles supplied soon led to an application on the part of others to participate the benefit. In a few years, consequently, from this time, the Company became a trading body, and supplied all customers.

In 1694, apothecaries were exempted from serving the offices of constables, scavengers, &c.; by this time they had increased so rapidly in numbers, as to have become a very influential body. By practising medicine as well as pharmacy they excited the jealousy of the physicians, between whom and the apothecaries a violent contest now rose; pamphlets were published on both sides, and the dispute ran high, till at last the physicians resolved to put down the apothecaries by establishing dispensaries, where they supplied medicines on reasonable terms, made up under their own superintendence.

The dispensaries prospered, but in process of time, the physicians becoming weary of the drudgery of the business, established the assistants whom they had employed and instructed at these places, as dispensing chemists on their own account; some of the apothecaries, finding the business profitable, adopted the same course, and from this we may date the origin of chemists and druggists.

In 1723, the College of Physicians were again empowered to visit the shops of apothecaries, and examine the drugs; this right however was not always exercised in a very impartial manner, nor indeed does it appear that the persons employed were always very competent.

Thus, an anecdote is related, that some of the examiners coming to an apothecary's shop, found a pot with *ung. album* written on it: the gentlemen got about the pot and examined it; each gave his opinion—one said it was hard, another that it did not smell enough of camphor, a third that it ought to be malaxed with some oil, while a fourth proposed treating it in a summary manner by throwing it out of doors. In the height of the dispute, the boy coming in, entreated them to stay their hands, assuring them that it was perfectly good, but only put into the wrong vessel. " What is it then?" said one of the learned. " Why, gentlemen," said the boy, " 'tis white dog's ——."

In the Pharmacopœia of 1746, a great improvement had taken place over that which preceded it, many of the complicated and absurd formulæ were removed, and considerable progress had been made in the chemical part. The Mithridatum with its forty ingredients, and Theriaca Andromachi with about sixty, were still however allowed to remain.

The origin of the Botanic Garden at Chelsea is involved in some obscurity, but it is supposed to have been founded prior to the year 1673. In 1722, Sir Hans Sloane, who had purchased the manor, granted it on lease to the Society of Apothecaries, on condition that they should present annually to the Royal Society fifty distinct plants, till the collection amounted to 2000, and also that the garden should be appropriated to the purpose of cultivating plants, instructing students, and advancing science.

Towards the latter end of the last century, disputes having arisen between the chemists and apothecaries, the latter formed themselves into a Society under the title of the General Pharmaceutical Association of Great Britain, for the purpose of preventing chemists from vending pharmaceutical preparations, compounding physician's prescriptions, &c. The result of their exertions, however, was not so successful as was anticipated, and the Society shortly broke up without having effected much against the druggists, whom they had threatened to destroy.

For the remainder of the account of the differences between the apothecaries and chemists and druggists, we must refer to the pamphlet itself, in which our readers will find much to instruct and amuse them.

---

REMARKS ON MEDICAL REFORM, IN A SECOND LETTER TO SIR JAMES GRAHAM, BART. &c. By Sir *James Clark*, Bart. Pp. 40. March, 1843.

THE dice are rattling in the box, and the next throw will probably decide the fate of MEDICAL REFORM—at least, for many years to come! We confess that the hollow sound of the dead bones vibrates rather ominously on our ears—and we should be less surprised than grieved if the dice turn up sixes and sevens instead of aces. Instead of having a united Board or Body—a "tria (or duo) juncta in uno"—to regulate and equalize the education of the profession in England, we shall perhaps have the DISJECTA MEMBRA brought forward, a little whitewashed and varnished, but still essentially the same—each pulling its own way, and for its own corporate interest. The time for appeal is short—and we regret to see that the zeal, the energy, and the union of the profession are far from corresponding to the emergency of the occasion! Sir James Clark and others, including ourselves, have urged on the public, for some time past, the necessity of petitioning the legislature on this important subject—and if active measures are not now quickly taken, the cause will be lost.

We made our readers acquainted with our author's former Letter to Sir James Graham. The present brochure briefly recapitulates the prominent features of the appeal, and adds fresh arguments and illustrations.

"My principal object in addressing you at present is to urge the necessity of so framimg the legislative enactment which you are about to introduce, as thereby to secure to all who are permitted to engage in the practice of medicine a GOOD EDUCATION. This is unquestionably the most important part of what is implied by medical reform, and that calling most loudly for the interference of the Legislature. Until it is obtained, any scheme of reforming or modifying the existing medical corporations will, in my belief, be productive of little benefit to the profession and less to the public."

Our author, as we ourselves have long done, urges the propriety of one uniform system of medical education for all classes, including the general practitioner, leaving those who choose to go beyond that *minimum* of acquirements to do so, if they please, and pursue any particular department of physic or surgery to which their genius or inclination prompts them. Such curriculum, he thinks, ought to be determined, not by any corporate body, but by a Board of Commissioners appointed by Government, and quite independent of the Colleges or the teaching corps.

Sir James dilates eloquently and urgently on the necessity of a far superior preliminary education—literary and scientific—to that which now obtains—if, indeed, anything of the kind now obtains at all. This course of study, he thinks, and we agree with him, should be *completed* before the professional courses commence. Such preliminary education will, in fact, facilitate the course of medical studies that are to follow. The apprenticeship-system he, of course, completely throws overboard, as it has now very few advocates.

As the general practitioner is now the general physician and surgeon of the public, and consequently has an overwhelming responsibility thrown on his shoulders, his education and examinations should be precisely the same as those who intend to pursue physic or surgery exclusively afterwards. The latter, as we before observed, may extend their studies and acquirements to any further amount they please.

In the following sentiments we entirely agree.

" Of the advantages which would accrue both to the profession and the public from the establishment of a better system of education throughout the empire for all medical practitioners, there will, I apprehend, be little difference of opinion. But upon the actual amount of that education, more especially as regards the general, or what has been termed the preliminary education, there may be much; —such difference, however, arising not from any want of agreement respecting the utility, or even necessity of such preliminary instruction, but upon other grounds. The principal of these grounds are, I believe, the following:—1st. The difficulty of obtaining the requisite preliminary education; 2nd, the want of means and time on the part of the student; 3rd, the notion that if much general education is called for, there may be a deficiency in the number of young men entering the profession; and 4th, the suspicion that men so educated would not undertake the drudgery of attending the poor.

" The first objection might have had some weight a few years ago, but has little or none at present, when public educational institutions, at which all the necessary instruction is given, are so general, that no town of any consequence is without one.

" The second objection is inadmissible. The want of means on the part of the student is not a legitimate reason for not requiring the necessary education; but it does afford a conclusive reason why a youth so circumstanced, should not enter the profession at all. The chief cause of the evils of which I complain has arisen from youths entering the profession totally deficient in preliminary education, and without the means of enabling them to obtain more than a *minimum* of medical education. Want of time will form no objection when the apprenticeship system is abolished; and I believe it has now scarcely an advocate. 'An apprenticeship may unhesitatingly be pronounced pernicious, which absorbs either the means or the time that ought to be devoted to the acquisition of preliminary and scientific knowledge. And when we remember the circumstances under which a large proportion of medical apprenticeships are at present passed, at a distance from any school where either preparatory or professional knowledge can be acquired, and in the performance of a perpetual routine of menial services, which could be performed with equal advantage to the public by the most uneducated,—such apprenticeships cannot but be considered as an arrangement in which the interests of those who are training to the medical profession are sacrificed to the interests of those who are already engaged in its practice.'* "

"The third objection to good preliminary education I consider of little weight. By requiring a higher standard of education, the profession would be made more respectable, and young men of a better class, and better educated, would be found in abundance, I doubt not, willing to enter it; and should it have the effect of diminishing the number of medical students, neither the profession nor the public would, I apprehend, be losers by such a result. At present, the medical profession is over-crowded; and if the enlightened views which are now being promulgated for improving the public health, more especially that of the working classes, are fully carried out, the present proportion of medical men, may, I believe, be considerably diminished with perfect safety to the public.

"With respect to the remaining objection—that well-educated young men would not submit to the drudgery of attending the poor—I believe it to be equally visionary with the others, and, at any rate, it will be time enough to legislate for such a case when it occurs."

In illustration of the great neglect of the medical student's preliminary education, our author quotes a remarkable passage from Mr. Guthrie's Lectures.

"I regret to say," observes that gentleman, "that among the students who entered the profession some years back, and are only *now* presenting themselves for examination under the regulations of 1836, there are many who cannot spell very common words in their native language."

This is a sad picture of the British Surgeoncy! The next great point urged by Sir James, and advocated for twenty years by ourselves, is the disjunction of pharmacy from physic. There is no necessary connection, *in practice*, between the two. Their union is a remnant of the dark ages—before the division of labour was advanced—and when every workman made his own tools. Who would not stare to see Sir Astley Cooper or Sir Benjamin Brodie, working half their time with their leather aprons and tucked up sleeves, constructing their gorgets, catheters, scalpels, lancets, catlins, and the whole of the armamentum chirurgicum!!

It is so with the physician and surgeon—and, *a fortiori*, with the "GENERAL PRACTITIONER," who has so many other irons in the fire. For although it is necessary for all three denominations to *study* pharmacy—it is totally unnecessary to practise it—except at the expense of other and more important avocations and investigations. But this is not the worst of the union. Pharmacy is a scientific *trade*—physic a liberal *profession*. The two should never be combined. As long as they are linked together, the public will look at the traffic in pills, potions, and draughts, with an eye of suspicion and distrust. Moreover, the general practitioner will never get the free, and unshackled, and undisputed power to charge for his *skill*, while he throws in *physic* at the same time.

But all these things must be urged—and quickly urged, on the notice of the Legislature by numerous and respectably signed petitions from every part of the kingdom. The letter of an individual to the Home Secretary, however distinguished that individual may be, will not counterbalance the ear-wigging of those whose interests are likely to be affected by any thorough reform in the profession. In the mean time we recommend the immediate perusal of both the Letters in question, as furnishing data, forms, and substance for petitions to both Houses of Parliament.

---

* Dr. Thomson: Life of Cullen, vol. i, p. 515.

On Diseases of the Bladder and Prostate Gland. With Plates. By *William Coulson*. Third Edition. 8vo. pp. 274. London: Longman and Co.

The appearance of a *third* edition of this work is good evidence of the estimation in which it is held, while the care with which each successive edition has been revised is also evidence that the author deserves the patronage he has experienced. Our opinion has been already expressed so freely in his favor that it is unnecessary to reiterate it, suffice it that we recommend the book strongly to the profession.

Mr. Coulson has seen no reason to alter his views on the subject of urinary diseases, nor the arrangement of his observations upon them. But he appeals to what has been added to the chapter on Urine, as well as to those on Stone and on the affections of the Prostate Gland, as proofs of the attention with which he has reconsidered his book.

The chapter on the Urine is indeed very complete, and merits careful perusal. Its chemical composition in health, its alterations in disease, the modes of examining it, and the indications to be drawn from it are set forth both with perspicuity and completeness.

The chapters on Stone too, are copious and accurate. The following is Mr. Coulson's plan of operating for the removal of the stone.

"I introduce a curved staff, and give it to the care of an assistant, directing the handle to be inclined a little towards the grouud, and the groove to be turned towards the left side. By this inclination of the handle, the groove of the staff is certainly made less prominent in the perineum; but there is this advantage attending it, that when we have cut into the groove, there is no occasion to alter the position of the staff, and the fore-finger of the left hand is quite at our disposal for protecting the rectum, and guiding the knife. I find that I can perform the operation much more rapidly in this way, than by taking the staff into my own hand. M. Langenbeck is a strong advocate for this mode of holding the staff; he, however, advises the handle to be inclined still more towards the ground than I do. I begin the first incision rather low, about two fingers' breadth above the anus: the bulb of the urethra will then be avoided. In fact, the external incision if commenced higher, can be of no use to the operator; and I find that this, the upper part of the wound, is often the slowest to heal. For the division of the prostate, I use the long straight knife, with the beak in the middle line of the point; and I feel confident that it is the safest and best instrument that can be employed."

He gives a caution with regard to the performance of lithotrity.

"Although lithotrity is often very easily performed, it is necessary that the patient's health should be brought into a favourable condition for the operation; the same care should be taken for a few days at least both before and after it as in other important operations. It has been too much the custom of late years to regard the operation of lithotrity as a light one, and to pay but little attention to the subsequent management of the patient. In one case in which I operated, the patient rode in a jolting vehicle from my house to Wandsworth, the consequence of which and of the operation (though it was performed without difficulty) was an attack of fever which confined the patient for some weeks. In another case, considerable hæmorrhage followed, which I attributed more to the exertion afterwards than to the operation itself. I have come, therefore, to the determination not to perform the operation of lithotrity except at the patient's house, and to keep him quiet for several days after each operation."

*Mode of Applying Leeches to the Prostate.*—Mr. Coulson refers to a plan employed by Mr. W. Craig in a case of diseased prostate. It consists of a tube into which is fitted a piece of wood, with a handle at one end, while the other terminates conically in a blunt point, for the purpose of gradually dilating the rectum. When this is effected, the wooden dilator is to be withdrawn, and a box of proper size to fit the tube, and capable of holding three or four leeches, with a piece of wire to form a handle fixed into the bottom of it, is to be pushed through the tube.

It is necessary to lubricate the wood and the tube with oil previous to using it, and in introducing it, it should be directed towards the rectum in order to prevent its coming in contact with the tender prostate.

*Application of Iodine to the Prostate.*—Mr. Coulson quotes Mr. Stafford's method of applying iodine to the gland. "A bougie," says Mr. S., "is to be charged at its point with the iodine, iodide of potassium, or any other substance you may wish, and then dipping it into melted tallow, so that a coating may be formed upon it: by such method, I have been enabled to introduce any application I might desire up to the prostate gland, without touching the surface of any other part of the urethra. The bougie having reached the desired spot, its point is allowed to rest upon the diseased part, when the tallow gradually melts, and brings the iodine or iodide of potassium into contact with it, and by drawing the bougie gently backwards and forwards, the necessary friction is produced. I have found it advisable to be very cautious as to the strength of the application, for the prostate gland will not bear a strong preparation either of the iodine or iodide of potassium at first. It is usually in an irritable or inflamed state, consequently, even the mechanical pressure of the bougie will give pain. The preparations I have therefore used have been very mild. At first I have found it necessary to employ even anodynes, such as belladonna, opium, hyoscyamus, &c. to quiet irritation and pain. When these have subsided, I have begun carefully by introducing the iodide of potassium, in the proportion of one grain to the drachm of unguentum cetacei, and increasing it as the patient could bear it; I have then gone on with two, three, four, five, and even as far as ten grains or a scruple to the drachm, according as the case required it. After this I have added iodine to it, half a grain, one, two, three, four, and even more grains in the same manner."

Again we beg to recommend the work.

